# Non-pharmaceutical interventions during the COVID-19 epidemic changed detection rates of other circulating respiratory pathogens in Japan

**DOI:** 10.1371/journal.pone.0262874

**Published:** 2022-01-21

**Authors:** Yuki Nagakubo, Yosuke Hirotsu, Makoto Maejima, Masahiro Shibusawa, Kazuhiro Hosaka, Kenji Amemiya, Hitomi Sueki, Miyoko Hayakawa, Hitoshi Mochizuki, Toshiharu Tsutsui, Yumiko Kakizaki, Yoshihiro Miyashita, Masao Omata

**Affiliations:** 1 Genome Analysis Center, Yamanashi Central Hospital, Kofu, Yamanashi, Japan; 2 Division of Microbiology in Clinical Laboratory, Yamanashi Central Hospital, Kofu, Yamanashi, Japan; 3 Division of Genetics and Clinical Laboratory, Yamanashi Central Hospital, Kofu, Yamanashi, Japan; 4 Central Clinical Laboratory, Yamanashi Central Hospital, Kofu, Yamanashi, Japan; 5 Department of Gastroenterology, Yamanashi Central Hospital, Kofu, Yamanashi, Japan; 6 Lung Cancer and Respiratory Disease Center, Yamanashi Central Hospital, Kofu, Yamanashi, Japan; 7 The University of Tokyo, Bunkyo-ku, Tokyo, Japan; Waseda University: Waseda Daigaku, JAPAN

## Abstract

Severe acute respiratory syndrome coronavirus 2 (SARS-CoV-2) has circulated worldwide and causes coronavirus disease 2019 (COVID-19). At the onset of the COVID-19 pandemic, infection control measures were taken, such as hand washing, mask wearing, and behavioral restrictions. However, it is not fully clear how the effects of these non-pharmaceutical interventions changed the prevalence of other pathogens associated with respiratory infections. In this study, we collected 3,508 nasopharyngeal swab samples from 3,249 patients who visited the Yamanashi Central Hospital in Japan from March 1, 2020 to February 28, 2021. We performed multiplex polymerase chain reaction (PCR) using the FilmArray Respiratory Panel and singleplex quantitative reverse transcription PCR targeting SARS-CoV-2 to detect respiratory disease-associated pathogens. At least one pathogen was detected in 246 (7.0%) of the 3,508 samples. Eleven types of pathogens were detected in the samples collected from March–May 2020, during which non-pharmaceutical interventions were not well implemented. In contrast, after non-pharmaceutical interventions were thoroughly implemented, only five types of pathogens were detected, and the majority were SARS-CoV-2, adenoviruses, or human rhinoviruses / enteroviruses. The 0–9 year age group had a higher prevalence of infection with adenoviruses and human rhinoviruses / enteroviruses compared with those 10 years and older, while those 10 years and older had a higher prevalence of infection with SARS-CoV-2 and other pathogens. These results indicated that non-pharmaceutical interventions likely reduced the diversity of circulating pathogens. Moreover, differences in the prevalence of pathogens were observed among the different age groups.

## Introduction

On March 11, 2020, the World Health Organization declared a worldwide pandemic of severe acute respiratory syndrome coronavirus 2 (SARS-CoV-2) [1]. As of October 2021, more than 230 million people have been infected with this virus, and 4.8 million have died as a result [2]. To suppress the spread of the virus, infection prevention measures have been implemented in many countries.

The “common cold” refers to mild upper respiratory illness characterized by symptoms such as nasal congestion and discharge, sneezing, sore throat, and cough [3]. Several types of viruses are associated with the common cold [3, 4]. Rhinoviruses are responsible for approximately 30%–50% of all colds, human coronaviruses (HCoVs) are responsible for approximately 10%–15%, and influenza viruses are responsible for approximately 5%–15%. The number of patients infected with influenza or HCoVs increases during winter [5–10]. Coronaviruses belong to the Coronaviridae family and the ones known to infect humans belong to two genera, α-coronaviruses (HCoV-229E, HCoV-NL63) and β-coronaviruses (linage A, HCoV-OC43, HCoV-HKU1; linage B, SARS-CoV-1, SARS-CoV-2, MERS-CoV).

We previously reported that SARS-CoV-2 was detected in Yamanashi, Japan in March to May 2020, whereas no patients were found to be infected with influenza viruses during this time [11]. Concordantly, the prevalence of influenza virus remained extremely low in Australia (March–September 2020) [12], the Southern Hemisphere (April–July 2020), and during the interseasonal circulation in the USA (May–August 2020) [13] compared with their typical rates before the coronavirus disease 2019 (COVID-19) pandemic [14]. These findings suggest that, along with influenza viruses, the prevalence of other respiratory-related viruses in the community had changed; however, this hypothesis has not been fully verified.

In this study, we examined the prevalence of circulating viruses and pathogens after the onset of the COVID-19 pandemic. To this end, we conducted a surveillance study by performing nucleic acid amplification tests to clarify the trends in the circulation of several types of respiratory viruses, including SARS-CoV-2, influenza viruses (A and B), common coronaviruses (HCoV-OC43, -229E, -NL63, and -HKU1), and other pathogens.

## Materials and methods

### Patients and samples

From March 1, 2020 to February 28, 2021, we collected samples from patients who visited Yamanashi Central Hospital, Japan. The majority of these patients showed at least one symptom of fever, headache, fatigue, nasal congestion, nasal discharge, sneezing, sore throat, and/or cough. Some asymptomatic individuals who had close contact with an individual infected with SARS-CoV-2 were also included. Individuals who were judged by doctors to need testing were included; there were no exclusion criteria.

We included a total of 3,249 patients (1,821 men and 1,428 women) in the study. The age ranged from 0 to 103 years old, with an average age of 58.6 years. In all, 3,508 nasopharyngeal swab samples were collected, because in some cases two or more samples were taken from the same patient at different time points. All samples were collected with cotton swabs and were stored in viral transport medium (Copan, Murrieta, CA, USA). In instances where the test failed to detect the internal controls or when the test results were not available, the samples were retested. The data was collected from the electronic records after testing.

The Institutional Review Board of Yamanashi Central Hospital approved this study, which complied with the Declaration of Helsinki and used the opt-out consent method with written notice for all patients (approval number: G-2019-1). This study used data obtained in the regular course of medical diagnosis, and no additional procedures were required of the patients during the study. To avoid identifying personal information, the results were obtained and analyzed from the data of a large number of people and do not include personal data. The requirement for written informed consent was waived because this was an observational study.

### FilmArray respiratory panel (RP)

We performed multiplex PCR targeting 18 viruses and three bacteria species using FilmArray RP v1.7 (bioMérieux, Marcy-l’Etoile, France) as previously described [11]. Briefly, buffer and 300 μL of viral transport medium were injected into the FilmArray pouch. The reaction proceeded automatically on the FilmArray Torch system [15]. We also used a newer version of FilmArray RP, v2.1 [16], which additionally targets SARS-CoV-2. We tested 370 samples using FilmArray RP v1.7 that were collected between March 1, 2020 and August 4, 2020 and 3,138 samples using FilmArray RP v2.1 that were collected between August 5, 2020 and February 28, 2021. If the internal positive control was not detected (failed or invalid), we used the same viral transport medium and retested the sample. FilmArray RP does not distinguish between rhinoviruses and enteroviruses. For this study, when a rhinovirus or enterovirus was detected, they were treated as a single pathogen for convenience.

### Viral nucleic acid extraction

The total nucleic acid was automatically isolated from the nasopharyngeal swabs using the MagMax Viral/Pathogen Nucleic Acid Isolation Kit (Thermo Fisher Scientific, Waltham, MA, USA) on a KingFisher Duo Prime system (Thermo Fisher Scientific) as previously described [17, 18]. Briefly, we added 200 μL of viral transport medium, 5 μL of proteinase K, 265 μL of binding solution, 10 μL of total nucleic acid-binding beads, 0.5 mL of wash buffer, and 0.5–1 mL of 80% ethanol to each well of a deep-well 96-well plate. The nucleic acids were eluted with 70 μL of elution buffer. The total nucleic acids were immediately subjected to quantitative reverse transcription PCR (RT-qPCR).

### RT-qPCR

To detect SARS-CoV-2, we performed one-step RT-qPCR in accordance with the protocol developed by the National Institute of Infectious Diseases in Japan [19]. This PCR method amplifies the nucleocapsid gene of SARS-CoV-2 (NC_045512.2) [17]. The reaction mixture was composed of 5 μL of 4× TaqMan Fast Virus 1-Step Master Mix (Thermo Fisher Scientific), 1.0 μL of 10 μM forward primer (5′-AAATTTTGGGGACCAGGAAC-3′), 1.4 μL of 10 μM reverse primer (5′-TGGCAGCTGTGTAGGTCAAC-3′), 0.8 μL of 5 μM probe (5′-FAM-ATGTCGCGCATTGGCATGGA-TAMRA-3′), 6.8 μL of nuclease-free water, and 5 μL of nucleic acid sample in a 20-μL total volume. The expected amplicon size was 158 bp. The human ribonuclease P protein subunit p30 (*RPP30*) gene was used as an internal positive control (Integrated DNA Technologies, Coralville, IA, USA) [17].

The RT-qPCR assays were conducted on a StepOnePlus Real-Time PCR system (Thermo Fisher Scientific) with the following cycle conditions: 50°C for 5 min for reverse transcription, 95°C for 20 s, and 45 cycles of 95°C for 3 s and 60°C for 30 s. The threshold was set at 0.2. In accordance with the national protocol (version 2.9.1) [19], samples were assessed as positive if a visible amplification plot was observed and assessed as negative if no amplification was observed.

From March 1, 2020 to August 4, 2020, we conducted RT-qPCR on samples; these were the same samples (described above) analyzed by FilmArray RP v1.7.

### Statistics

Pearson’s chi-square test with cross-tabulation was performed to determine the pathogen infection status by age group. P-values were computed by Monte Carlo simulation.

## Results

### Analyzed samples

From March 1, 2020 to February 28, 2021, a total of 3,508 nasopharyngeal swab samples were collected from 3,249 patients (Table 1 and S1 Fig). An average of 292 samples were tested each month (range: 36–696) (S1 Fig). All samples were subjected to multiplex PCR testing (FilmArray RP v1.7 or v2.1) to identify the presence of respiratory pathogens [20]. We also tested for the presence of SARS-CoV-2 using RT-qPCR as previously described [17, 18]. Over the study period, we identified 246 samples that were positive for at least one pathogen. This accounted for 7.0% of all samples (246/3,508).

**Table 1:** Age distribution of the 3,508 samples.

Age (year)	Number of samples	(%)
0–9	268	7.7
10–19	133	3.8
20–29	282	8.1
30–39	219	6.3
40–49	261	7.5
50–59	310	8.9
60–69	376	10.7
70–79	703	20.1
80–89	701	20.0
90–99	247	7.1
≥100	8	0.2
**Total**	**3,508**	**100**

### Emergency declarations and pathogen detection after onset of the COVID-19 pandemic

**Fig 1:**
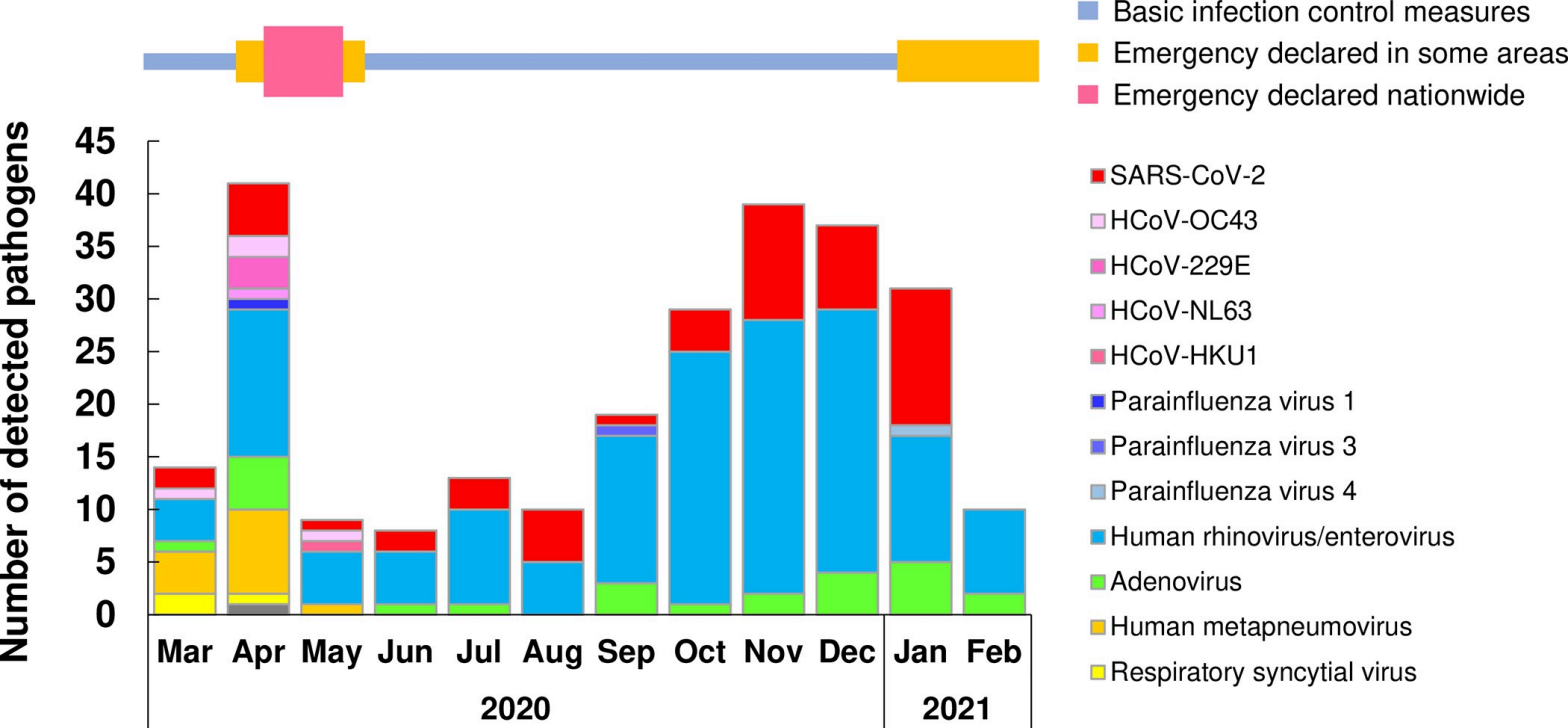
Trends in the circulation of SARS-CoV-2 and other respiratory pathogens during the COVID-19 pandemic.

We examined whether the restrictions imposed by the emergency declaration changed the types of pathogens detected. In February 2020, the Japanese government declared COVID-19 as a "designated infectious disease" and recommended that basic infection control measures be implemented nationwide (e.g., wearing masks, hand washing and sanitizing, and keeping a safe distance between people) (Fig 1). Subsequently, the first emergency declaration was issued in the beginning of April for some areas; this emergency declaration was then expanded to all of Japan (including Yamanashi Prefecture) on April 16, 2020, and was in place until May 14, 2020 (Fig 1). The emergency declaration requested people to refrain from going out, closed schools, recommended telework, and restricted the use of facilities where people gather. A second emergency declaration was implemented from January 8 to March 21, 2021 in neighboring prefectures but did not include Yamanashi Prefecture (Fig 1). The second emergency declaration requested restaurants to shorten their hours and asked people to refrain from traveling to the designated areas.

The schematic at the top of the figure depicts the timing of basic infection control measures and emergency restrictions implemented by the Japanese government. The bar plot shows the monthly number of respiratory pathogens detected during the study period. Each color indicates a different virus type.

From March to May 2020, we tested 292 samples and found 59 positive samples (20.2%) and 233 negative samples (79.8%). Eleven types of pathogens were identified (Fig 1). During this period, we detected human rhinovirus / enterovirus (n = 23), human metapneumovirus (n = 13), SARS-CoV-2 (n = 8), adenovirus (n = 6), HCoV-OC43 (n = 4), HCoV-229E (n = 3), respiratory syncytial virus (n = 3), HCoV-NL63 (n = 1), HCoV-HKU1 (n = 1), parainfluenza virus 1 (n = 1), and *Mycoplasma pneumoniae* (n = 1). After June 2020, we tested 3,216 samples and found 187 positive samples (5.8%) and 3,029 negative samples (94.2%). However, only five types of pathogens were detected (Fig 1). The majority of samples in this period (98.9% [185/187 samples]) were positive for SARS-CoV-2 (n = 47), human rhinovirus / enterovirus (n = 128), or adenovirus (n = 19) (Fig 1). The other samples were positive for parainfluenza virus 3 (n = 1) or parainfluenza virus 4 (n = 1). These results suggested that the diversity of pathogens markedly fell after June 2020. The number of detected pathogen types decreased after the end of the first emergency declaration, when strong restrictions were imposed nationwide. However, few significant changes were seen before and after the second emergency declaration.

### Trends in pathogen detection during the observation period

**Fig 2:**
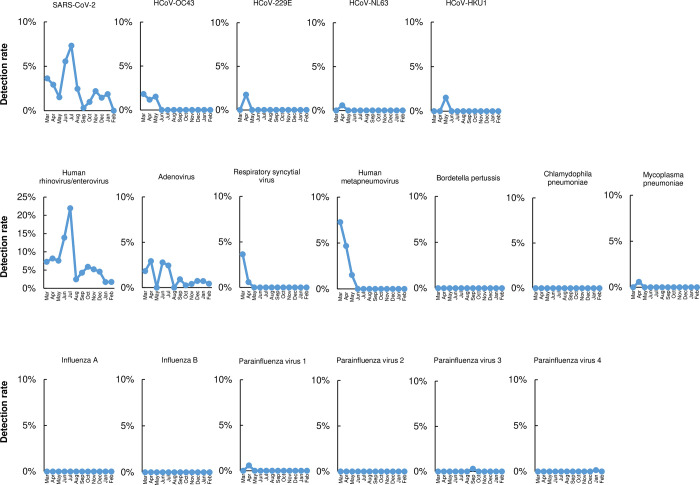
Detection frequency of each pathogen.

We next examined the detection rate of pathogens by month. SARS-CoV-2, human rhinovirus / enterovirus, and adenovirus were more prevalent than the other pathogens during the observation period (Fig 2). The months with the highest detection rates were July (7%, 3/41) for SARS-CoV-2, July (22%, 9/41) for human rhinovirus / enterovirus, and April (3%, 5/171) and June (2%, 1/36) for adenovirus (Fig 2). Human metapneumovirus and respiratory syncytial virus respectively peaked at 7% and 4% in March and decreased thereafter (Fig 2). Seasonal coronavirus was detected in 1% to 2% of samples throughout March to May but was not detected thereafter (Fig 2). We did not detect any influenza A virus or influenza B virus during the study period (Fig 2).

The detection rates were calculated by dividing the number of instances of each detected pathogen by the total number of pathogens tested in each period.

### Age distribution of infections

**Fig 3:**
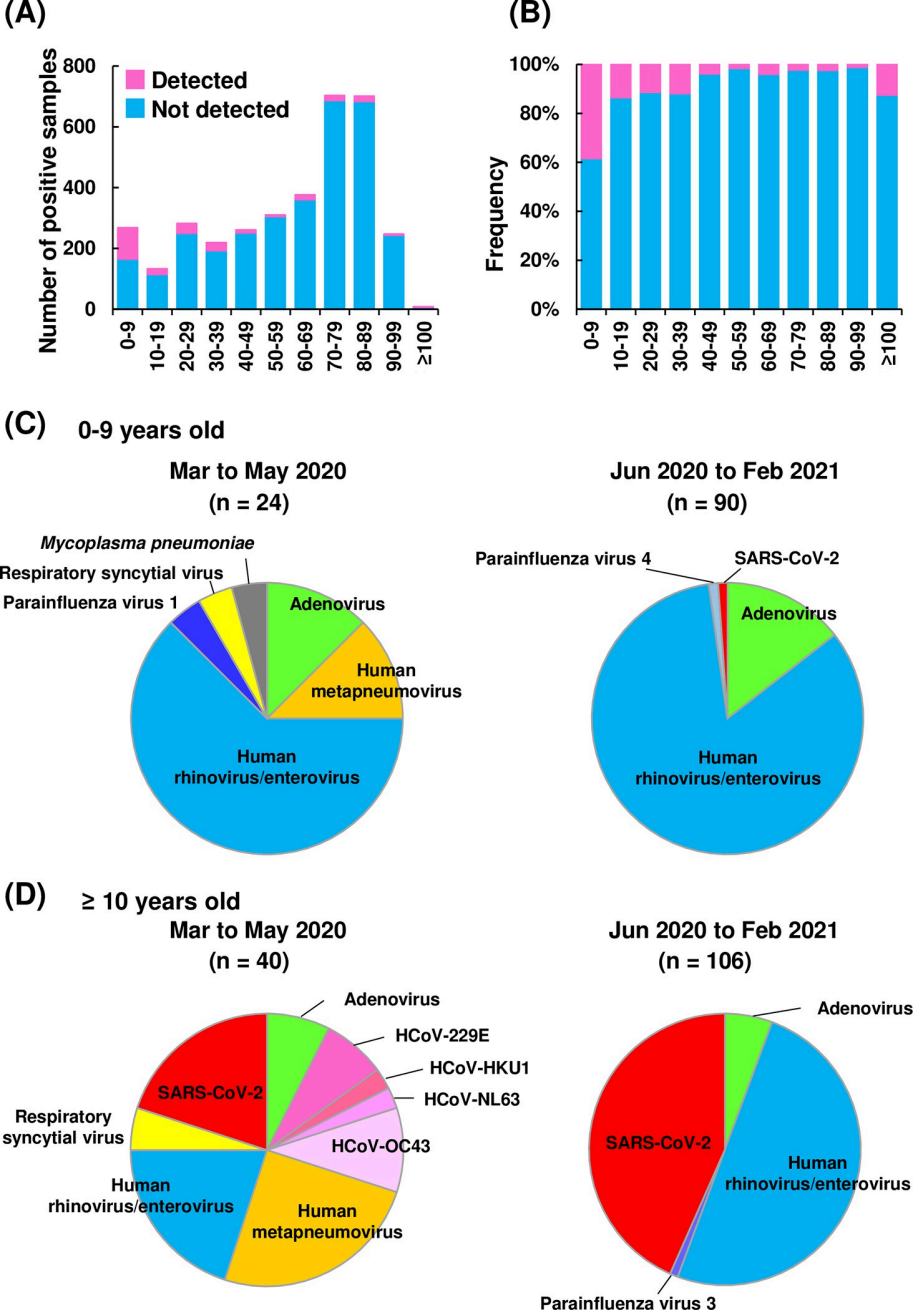
Respiratory-related pathogen infections by age group. **(A)** Number of positive samples in each age group. Bar plots indicate samples in which at least one pathogen was detected (pink) and samples in which none were detected (light blue). **(B)** Percentage of positive samples in each age group. The graph shows the number of positive samples detected in each age group divided by the total number of samples tested. The percentages were calculated based on the data in (A). **(C, D)** Pie charts show the pathogens detected during the period of March to May 2020 and June 2020 to February 2021. Age group represents data from 0–9 years (C) and 10 years and older (D). The number of positive samples were noted above the pie chart.

Previous reports have shown that the number of human rhinovirus detections in children (0–9 years) increased after the onset of the COVID-19 pandemic [21, 22]. Therefore, we analyzed whether there were differences in the pathogens detected in each age group. The samples analyzed were mostly from older adults (703 samples from those 70–79 years, 701 samples from those 80–89 years) (Fig 3A). The number of samples that were positive for at least one pathogen was highest in the 0–9 year age group (38.4%, 103/268), followed by the 10–19 year group (13.5%, 18/133), the 20–29 year group (11.3%, 32/282), and the 30–39 year group (11.8%, 26/219) (Fig 3A and 3B). Although the number of samples analyzed was small, the percentage with at least one pathogen detection was also high in those over 100 years old (12.5%, 1/8) (Fig 3A and 3B). Adenovirus and human rhinovirus / enterovirus were more prevalent in the 0–9 year group than in those older than 10 (S2 Fig and Table 2, p < 0.001, adenovirus; p < 0.001, human rhinovirus / enterovirus). SARS-CoV-2 and other pathogens were frequently detected in those older than 10 (S2 Fig and Table 2, p < 0.11, SARS-CoV-2; p < 0.006, Others).

**Table 2:** Distribution of detected pathogens by age.

Age (year)	Adenovirus (%)		Human rhinovirus/enterovirus (%)		SARS-CoV-2 (%)		Others (%)	
0–9	16	(64.0)	90	(59.6)	1	(1.8)	7	(24.1)
10–19	1	(4.0)	10	(6.6)	7	(12.7)	1	(3.4)
20–29	3	(12.0)	10	(6.6)	16	(29.1)	4	(13.8)
30–39	1	(4.0)	14	(9.3)	8	(14.5)	3	(10.3)
40–49	0		7	(4.6)	2	(3.6)	1	(3.4)
50–59	0		2	(1.3)	2	(3.6)	1	(3.4)
60–69	0		7	(4.6)	6	(10.9)	2	(6.9)
70–79	2	(8.0)	6	(4.0)	5	(9.1)	3	(10.3)
80–89	2	(8.0)	5	(3.3)	5	(9.1)	6	(20.7)
90–99	0		0		2	(3.6)	1	(3.4)
≥ 100	0		0		1	(1.8)	0	
**Total**	**25**		**151**		**55**		**29**	

SARS-CoV-2, severe acute respiratory syndrome coronavirus 2.

Present study showed the diversity of detected pathogens was reduced after June 2020 (Fig 1). To further analyze whether the number of pathogens reduced depending on the age distribution, we examined the relationship between the pathogens detected in each month and age groups. In 0–9 year group, six types of pathogens were identified during March-May 2020 and four types were identified during June 2020 to February 2021 (Fig 3C and S3A Fig). In age group older than 10, nine types were identified during March-May 2020 and four types were identified during June 2020 to February 2021 (Fig 3D and S3B Fig). Of note, human metapneumovirus and respiratory syncytial virus did not detect after June 2020 in both age groups. Common coronaviruses such as HCoV-229E, HCoV-HKU1, HCoV-NL63 and HCoV-OC43 were detected particularly in older than 10 years group in March-May 2020, but did not detect after June 2020. SARS-CoV-2 was observed in March-May 2020, but increased after June 2020 in the group of older than 10. These results indicated that the proportion of infectious pathogens varied by age group and thatthe diversity of detected pathogens was decreased regardless of age.

## Discussion

In this study, we analyzed the prevalence of respiratory pathogens, including SARS-CoV-2, in 3,508 samples over the first year of the COVID-19 pandemic. Several different types of pathogens were detected before May 2020. However, a smaller variety of pathogens was detected in the period from June 2020 to February 2021. This decrease in the number of pathogen types occurred in 0–9 and ≥10 year age groups. During the observation period, adenovirus, human rhinovirus / enterovirus, and SARS-CoV-2 were frequently detected. Adenovirus and human rhinovirus/enterovirus tended to be more prevalent in young children aged 0–9 years, which was consistent with previous reports [21, 22]. SARS-CoV-2 infection was more common in people aged 10 years and older, suggesting that there are differences in the types of pathogens that are easily transmitted by different age groups.

The preventative measures taken to mitigate the COVID-19 pandemic may have considerably changed the circulation of respiratory-rerated pathogens. Our data showed that the pathogen detection rates before and after the emergency was declared were markedly different (Fig 2). In Japan, a few months into the COVID-19 pandemic, more people started to wear masks, wash their hands more frequently, maintain physical distance, work from home, and restrict their travel [23]. Wearing a surgical face mask is effective for preventing the spread of viruses [24, 25]. This is likely because wearing a mask reduces the distribution of respiratory droplets that contain viral particles, thereby reducing the risk of infection [26].

Behavioral changes arising from the psychological reaction to a new infectious disease may have altered the infection situation. It is believed that people take action when they perceive a threat as serious and when the action is effective in reducing risk [27]. Big data analysis of mobile phone location data showed that the number of visitors from outside a prefecture was reduced by more than 90% compared with the previous year in tourist areas across Japan [28]. In Yamanashi Prefecture, the number of visitors decreased by 72%–84%. The dissemination of COVID-19-related information and the government recommendations raised awareness among individuals and motivated them to implement preventative measures [29]. The emergency declarations encouraged people to use non-pharmaceutical interventions such as wearing masks, hand disinfection, and social distancing. Such declarations may have served as an incentive for people to take personal measures, thus altering the prevalence of expected pathogens when compared with previous years.

However, we also found that some pathogens (adenovirus, human rhinovirus / enterovirus) continued to be detected during the observation period. These viruses are non-enveloped viruses that do not have lipid membranes and are more resistant to alcohol than enveloped viruses are [21]. It can be presumed that the effectiveness of hand disinfection with alcohol, one of the infection control measures, was low for these viruses.

This study had some limitations. First, FilmArray was the only method used to test for respiratory pathogens. Second, some factors can cause a negative result, such as the sampling procedure or PCR inhibitors or contaminants in the specimen; furthermore, pathogens may have been below the detection limit if the patient was recovering from an infection. Third, this study was conducted at a single center in Yamanashi Prefecture, which led to bias in the choice of sampling.

In conclusion, this study provides preliminary data on trends in circulating respiratory viruses during the first part of the COVID-19 pandemic. Additional epidemiological surveillance of circulating respiratory pathogens during the ongoing COVID-19 vaccination rollout will be necessary to fully understand virus prevalence trends throughout the COVID-19 pandemic.

## Supporting information

S1 Fig Number of tests by month. The number of samples tested each month throughout the study period. A total of 3,052 samples were analyzed.
(TIFF)

S2 Fig Number of representative pathogens was detected by age group. Number of positive detections in the 0–9 year group (orange) and 10 years and older group (blue). The separate bar plots show the number of samples positive for adenovirus, human rhinovirus / enterovirus, SARS-CoV-2, and other pathogens.
(TIFF)

S3 Fig Pathogens detected by age group between March 2020 and February 2021. **(A, B)** The bar plot shows the pathogens detected in each month by age group. Each age group represents data from 0–9 years (A) and 10 years and older (B). The graphs show the number of pathogens detected in each month (left panel) and the percentage of detected pathogens (right panel).
(TIFF)
