# Supplementary figures and images for: Non-pharmaceutical interventions during the COVID-19 epidemic changed detection rates of other circulating respiratory pathogens in Japan
Source: PLoS One. 2022 Jan 21;17(1):e0262874. doi: 10.1371/journal.pone.0262874 (PMC8782330; doi:10.1371/journal.pone.0262874)

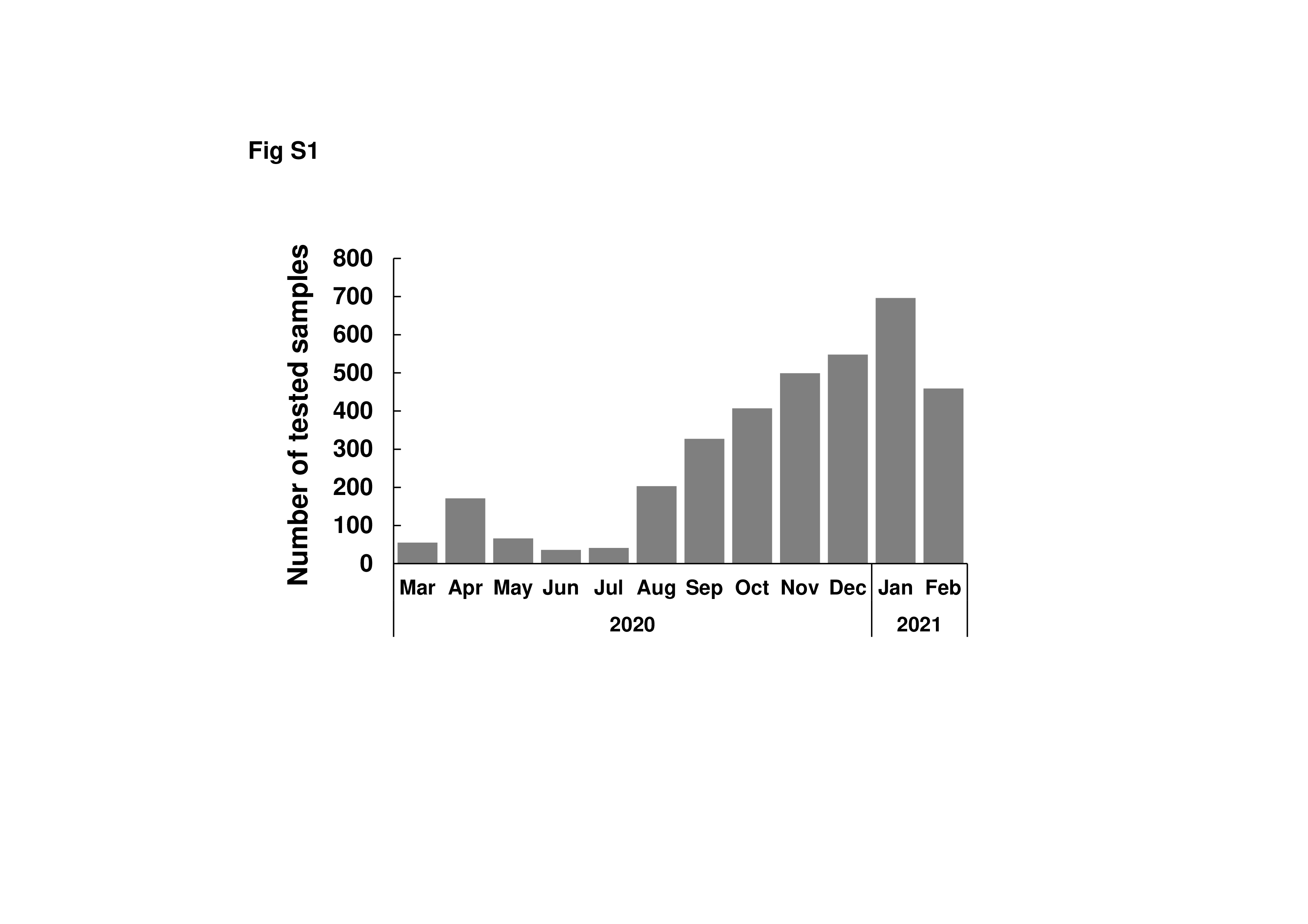

Supplement: S1 Fig — The number of samples tested each month throughout the study period. A total of 3,052 samples were analyzed. (TIFF) [file pone.0262874.s001.tiff]

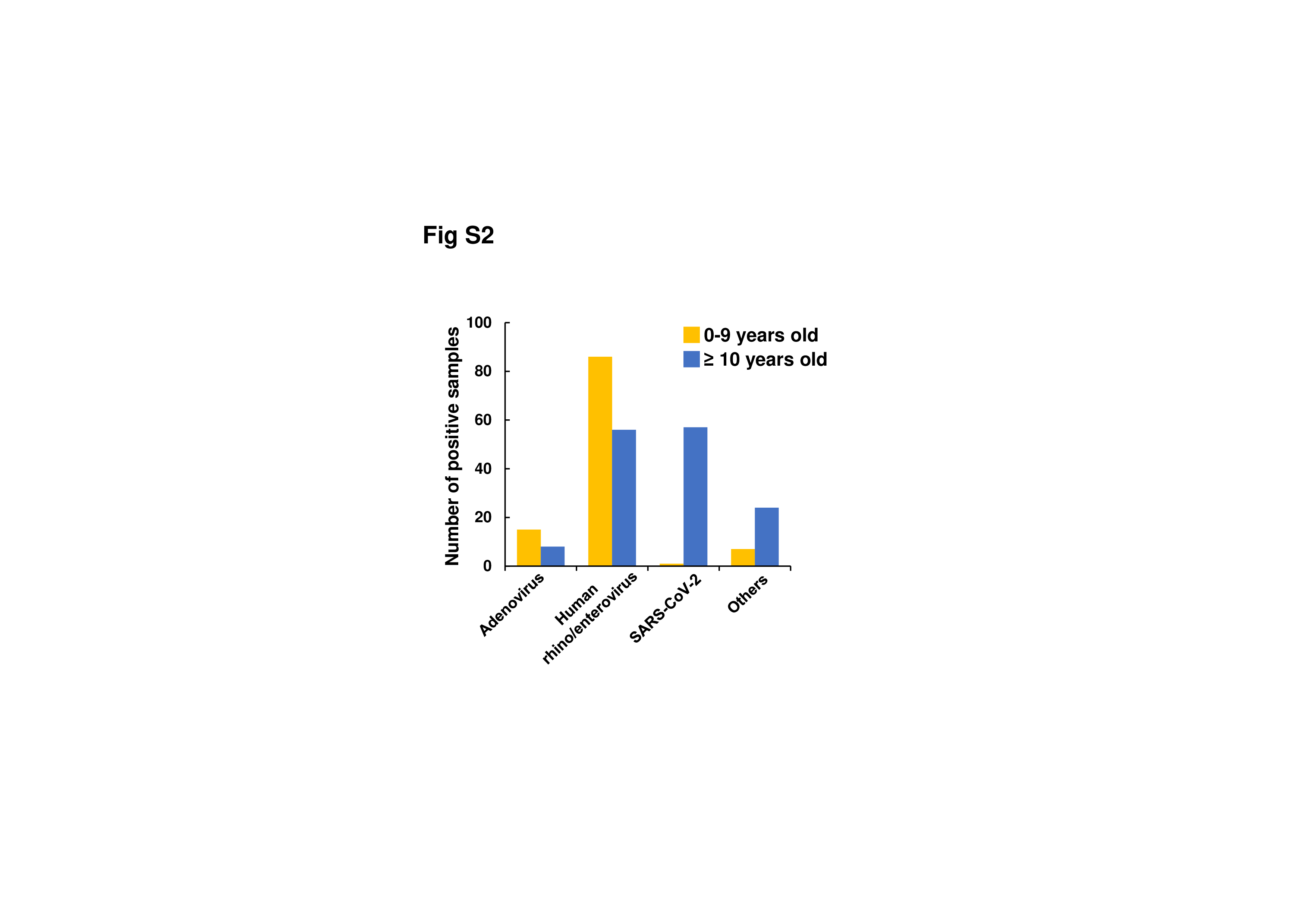

Supplement: S2 Fig — Number of positive detections in the 0–9 year group (orange) and 10 years and older group (blue). The separate bar plots show the number of samples positive for adenovirus, human rhinovirus / enterovirus, SARS-CoV-2, and other pathogens. (TIFF) [file pone.0262874.s002.tiff]

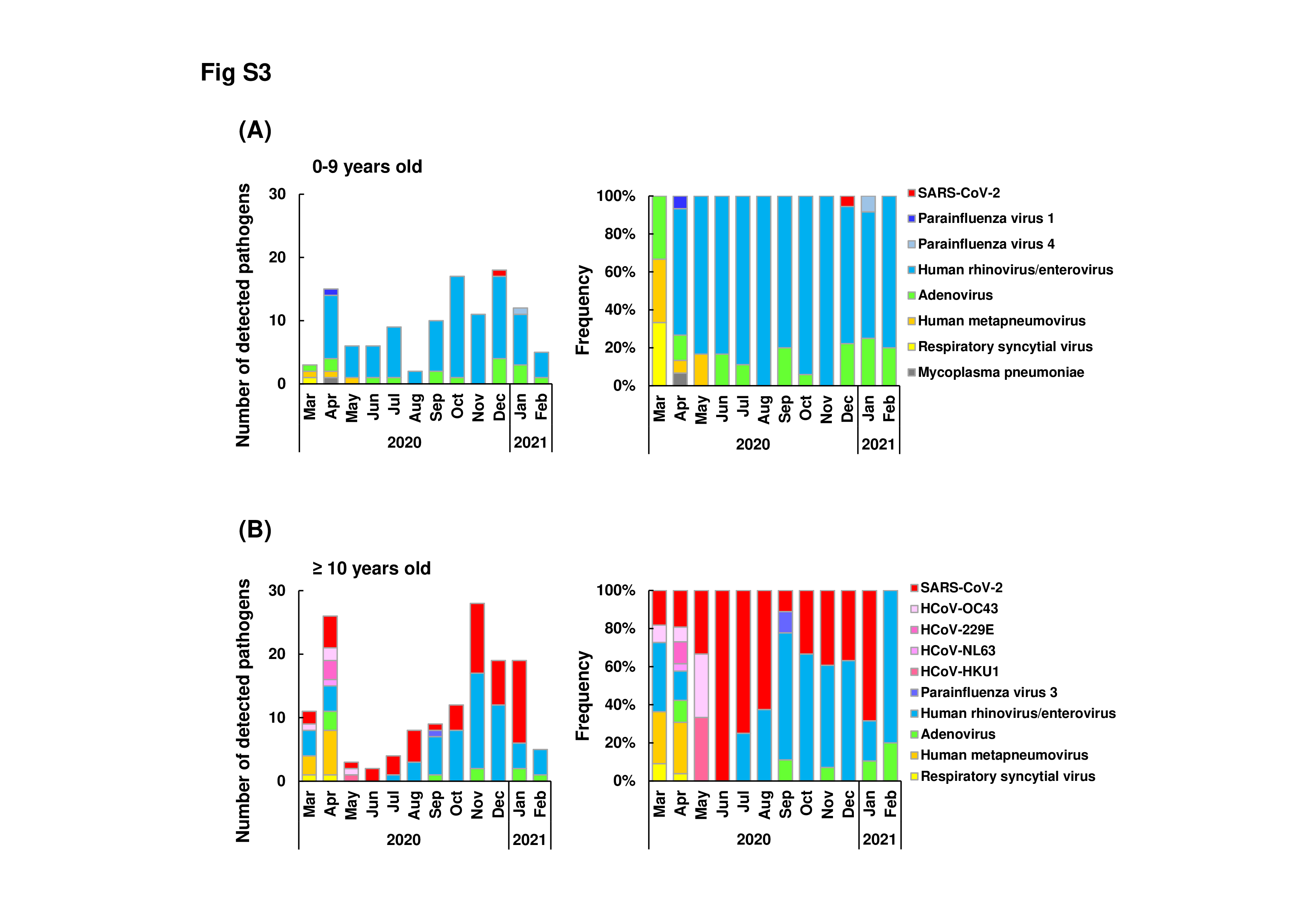

Supplement: S3 Fig — (A, B) The bar plot shows the pathogens detected in each month by age group. Each age group represents data from 0–9 years (A) and 10 years and older (B). The graphs show the number of pathogens detected in each month (left panel) and the percentage of detected pathogens (right panel). (TIFF) [file pone.0262874.s003.tiff]
